# What does it take to be rigid? Reflections on the notion of rigidity in autism

**DOI:** 10.3389/fpsyt.2023.1072362

**Published:** 2023-02-13

**Authors:** Valentina Petrolini, Marta Jorba, Agustín Vicente

**Affiliations:** ^1^Linguistics and Basque Studies, University of the Basque Country, Vitoria-Gasteiz, Spain; ^2^Department of Humanities, Pompeu Fabra University, Barcelona, Catalonia, Spain; ^3^Linguistics and Basque Studies, Ikerbasque Foundation for Science/University of the Basque Country, Vitoria-Gasteiz, Spain

**Keywords:** Flexibility, rigidity, autism, cognitive flexibility, Restricted Interests/Repetitive Behaviors

## Abstract

Characterizations of autism include multiple references to rigid or inflexible features, but the notion of rigidity itself has received little systematic discussion. In this paper we shed some light on the notion of rigidity in autism by identifying different facets of this phenomenon as discussed in the literature, such as fixed interests, insistence on sameness, inflexible adherence to routines, black-and-white mentality, intolerance of uncertainty, ritualized patterns of verbal and non-verbal behavior, literalism, and discomfort with change. Rigidity is typically approached in a disjointed fashion (i.e., facet by facet), although there are recent attempts at providing unifying explanations. Some of these attempts assume that the rigidity facets mainly relate to executive functioning: although such an approach is intuitively persuasive, we argue that there are equally plausible alternative explanations. We conclude by calling for more research on the different facets of rigidity and on how they cluster together in the autistic population, while suggesting some ways in which intervention could benefit from a finer-grained view of rigidity.

## Rigidity and the autism spectrum

Rigidity is often mentioned as a typically autistic trait in research, intervention and education, as well as in clinical practice (1–5). Yet, the notion of rigidity itself has not been the focus of detailed scrutiny, and normally encompasses several aspects that are not clearly distinguished from one another or further examined in their relation. In this paper we distinguish several notions of rigidity as discussed in the literature on autism,^1^ suggesting that they can be regarded as facets of a global rigidity trait. We also show that some of these facets are in principle dissociable from one another, and we call for more research along three main avenues: (i) identification and independent characterization of the different facets of rigidity; (ii) research on how often different facets come together in the autistic population; and (iii) alternative explanations of why these facets tend to cluster together more often in autistic people with respect to other populations. Concerning the latter point, we criticize an assumption that is at times made in the literature on the topic, according to which rigidity should be understood as an expression of cognitive inflexibility. We focus on this kind of approach because we have found it to be often taken for granted in the (relatively) scarce research on rigidity. Although we recognize its intuitive pull, discussing this assumption also serves to show that we are still far from understanding rigidity in autism satisfactorily. Intervention and diagnosis will be greatly improved if the rigidity construct is better understood and made more precise.

References to rigidity as a clinical feature are found in the description of autism in the DSM-5-TR (under ‘Autistic Spectrum Disorder’) and include stereotyped or repetitive movements or speech, insistence on sameness, inflexible adherence to routines, ritualized patterns or verbal non-verbal behavior, rigid thinking, and highly restricted or fixed interests. These different facets of rigidity, along with others, have a profound impact on day-to-day social interactions and often affect school or work performance as well as wellbeing.

Yet, as we mention above, there is still little agreement on how to think about rigidity in autism. This applies both to the search for a basic explanatory factor, if any, that may account for the observed patterns of behavior and thinking, and to the identification of specific facets of rigidity that caregivers and clinicians should be concerned with. Some researchers, for instance, focus on problems related to strict adherence to routines and the need for a structured learning environment, which they link to intolerance of uncertainty (3). Others discuss several facets of rigidity—e.g., insistence on sameness, restricted interests, and resistance to change—but fail to include intolerance of uncertainty (4). Legalism and literalism are further facets of rigidity that are often overlooked but may give rise to significant difficulties in educational settings. For instance, some participants in a recent study conducted by Wood and Happé (6) underscore specific difficulties in navigating “unspoken social rules”, “unspoken agreements”, and school politics more generally (p. 10). Overall, researchers agree that the construct of rigidity is currently too understudied and underdeveloped to represent a meaningful treatment target (4).

## Unpacking rigidity

Different aspects of rigidity are routinely assessed and measured in clinical settings through diagnostic tools, e.g., Autistic Diagnostic Observation Schedule-2 [ADOS-2—(7)]; Autism Diagnostic Interview-Revised [ADI-R—(8)]. Some scales, such as Strang et al.’s Flexibility Scale (5), are also employed for the same purpose, while others measure specific aspects of flexibility [e.g., the Interests Scale—(9); the Repetitive Behavior Scale-Revised—(10)]. Yet other scales are employed to assess flexibility in connection with executive functioning abilities—e.g., the Behavioral Rating Scale of Executive Function [BRIEF, (11)]; the Behavior Flexibility Rating Scale-Revised [BFRS-R; (10)].

Strang et al. (5) and Ozsivadjian et al. (2), which we discuss in more detail in the next section, are among the few works explicitly attempting to deal with rigidity as a uniform trait, in both cases reducible to cognitive inflexibility. Otherwise, we found a disjointed approach to rigidity in the literature, where different facets are discussed without any attempt at unifying them. An example of such disjointed approach is the widespread notion of “Restricted and Repetitive Behavior or Interest” (RRB) (12), which includes reference to several facets such as repetitive behavior (e.g., stimming or echolalia), insistence on sameness (e.g., lining up objects in a particular way), narrow and intense interests, and resistance to change (e.g., trouble with changing activity). Similarly, the ADOS-2 and the ADI-R include various items corresponding to rigidity features such as repetitive and stereotyped behaviors, compulsions and rituals, echolalia, circumscribed and unusual interests, and so on. Probably given their practical aims, neither of these diagnostic tools relates different rigidity facets to each other, nor they discuss them as different aspects of the same underlying construct.

Both ways of approaching rigidity, uniformly and disjointedly, strike us as problematic. On the one hand, we cannot simply assume that one notion of rigidity would encompass or explain all rigidity facets on the autism spectrum. In fact, we show that some facets of rigidity can be conceptually dissociated, and that there is some preliminary evidence in support of such dissociations. On the other hand, we cannot fail to notice that different facets of rigidity cluster together in some populations, and it is therefore necessary to discuss possible alternative explanations behind this fact. In what follows we introduce some of the ways in which autistic people are said to be rigid, according to the different notions existing in the literature as well as to first-person accounts.

*Fixed/restricted/special interests:* Special interests have been attributed to autistic individuals by a number of researchers (13–15). Turner-Brown et al. (16), for instance, show that interests in autistic individuals differ both qualitatively and in terms of intensity from interests of non-autistic individuals.

*Insistence on sameness and routines/rituals:* Insistence on sameness (IOS) is a construct originally introduced by Kanner (13), who connected this notion with restricted and repetitive behaviors as well as with anxiety, as he characterized autistic people as being driven by the “anxiously obsessive desire for the maintenance of sameness” (p. 245). In the recent literature, a distinction has been drawn between cognitive IOS and Repetitive Sensorimotor Behaviors. According to Bishop et al. (12), the latter include motor mannerisms, sensory-seeking behaviors, and repetitive use of objects, while IOS encompasses difficulties with changes in routines, compulsions, and rituals.^2^

*Intolerance of uncertainty (IU):* This construct has been characterized as a difficulty to deal with ambiguity or uncertainty in various aspects of life, or as the tendency to react negatively to uncertain situations and events (17). The expression is usually employed to encompass aspects related to discomfort with change and unknown situations, both in non-autistic and autistic populations ((18); see (19) for a recent criticism of the notion as applied to autism). IU has also been connected with higher levels of social anxiety in autism (20).^3^

*Black-and-white mentality:* This notion typically refers to being extreme in evaluations of people and actions, or to a more general inability to see nuances and gray areas in one’s own or other people’s thinking or behavior. Although there seems to be no empirical studies specifically devoted to black-and-white mentality in autism, and none of the scales we discuss here measures it directly, the construct comes up quite often in first-person accounts.^4^

*Strict adherence to rules:* This is a prototypical case of rigidity that refers to the intolerance of exceptions to rules, be they moral (e.g., one should not hurt others) or conventional (e.g., one should not eat on the floor). Although there is little research on this facet of rigidity, some studies on the moral/conventional distinction suggest that autistic people are stricter than non-autistic people when it comes to tolerating exceptions to rules (21). This facet of rigidity also appears in the Flexibility Scale developed by Strang et al. (5) under “Rigid about rules; legalistic”.^5^

*Literalism:* This facet of rigidity refers to the observed bias toward understanding metaphors, idiomatic expressions, implications, irony, and other figurative uses of language in a literal way (22). Although there is much research about literalism in autism, this facet of rigidity does not feature in typical diagnostic tools.

*Weak central coherence*: According to the weak central coherence account [originally proposed by Happé and Frith (23) and later re-labeled “local processing bias” by Booth and Happé (24)], autistic people experience difficulties in grasping the gist of something (e.g., a picture, a narrative) because they are absorbed by the details. Flexible thinking seems to involve moving beyond the particulars of a situation, finding analogies and ways in which things could have been different. Related to this, Klinger and Dawson (25) observe that autistic individuals often encounter issues in generalizing information to contexts other than the one in which the information was first presented.^6^

*Task-switching:* Task-switching is usually understood as the ability to shift between different thoughts or actions depending on the situational demands (26). As we explain below, task-switching is often interpreted as a key aspect of cognitive flexibility, a core component of the executive system that has been said to be disrupted in autism for a long time (27). Issues with task-switching result in perseveration errors or the repetition of the same response despite varying stimulus, or more generally in the inability to disengage from irrelevant tasks to engage in relevant ones (28).^7^

This preliminary characterization of rigidity shows that there are many ways of behaving, typical of autistic people (as well as of non-autistic people in various circumstances), which are described as rigid or inflexible in the literature. Each of them resonates with the others, but they are not *prima facie* identical or obviously reducible to one another, which in turn suggests that we may better conceive of rigidity as a *multifaceted* construct. However, the attempts at dealing with rigidity in a unified way often boil down to seeing all these facets as expressions of executive function problems, and of cognitive inflexibility in particular (2, 5).

In the next section we explore this guiding hypothesis, which we call “cognitive flexibility-first” (or “CF-first”) approach, focusing in particular on Strang et al.’s Flexibility Scale, since it is the most ambitious and comprehensive attempt at operationalizing the rigidity construct. Although it is indeed intuitive to think about rigidity in terms of executive function and cognitive flexibility, we offer some reasons to see this approach as resting on some unmotivated assumptions, while alternative approaches to rigidity are both available and worth pursuing. We show how the CF-first guiding hypothesis does not stand up to scrutiny once we analyze the more recent and comprehensive studies on rigidity in the autism spectrum. We then move on to highlight the more complex nature of the construct under discussion. This analysis of the CF-first approach also helps us to understand the rigidity construct better, as well as to illustrate ways in which future research can improve.

## More than one way of being rigid: Cognitive flexibility and the CF-first approach

Cognitive flexibility (CF) is characterized in various ways in the literature: as the ability to switch between discrepant tasks and demands [what we previously called “task-switching”, see Leung and Zakzanis (29)], or as the readiness with which one can switch between mental processes to generate appropriate responses (30). It is important to note that cognitive flexibility builds on other executive function processes like working memory and inhibition (31). Thus, it is an umbrella construct also measured by broader executive functioning assessments—such as the BRIEF—as well as by more specific tasks such as the Wisconsin Card Sorting Task, Luria’s hand sequence, Day/Night tasks, and many others. These tasks are usually structured so that subjects first familiarize themselves with one way of doing things, and then are asked to change it more or less abruptly. The recent literature on autism has approached CF mostly through the task-switching paradigm (26, 29, 32). However, Geurts et al. (26) show that current measures of CF are often insufficient to capture the complexity of factors affecting behavioral flexibility, that is the ability to adapt to different contexts and tasks in real life situations.

Also adopting a broader view of CF to include aspects of behavioral flexibility, Strang et al. (5) developed the Flexibility Scale (FS) as a multidimensional measure to capture flexibility as “a core component of executive function” (p. 2502). To our knowledge, this is the first attempt at studying rigidity in autism through a global and comprehensive approach. Strang and colleagues extracted five factors as a result of their analysis: Routines/rituals, Transitions/change, Special Interests, Social Flexibility, and Generativity. Routines/rituals include items such as “Does something special around bedtime” and “requires specific routes to familiar destinations”. Transitions/change mostly covers items related to inflexible behavior in response to change (e.g., “Complains when asked to do things differently”). The Special Interests factor touches upon intensity and interference with social life (e.g., “special interests interfere with conversation”). Social Flexibility includes a few reverse score items—such as “is a good sport” —as well as direct ones—e.g., “gets upset when losing a game”. Finally, Generativity mostly indicates one’s ability to come up with new ideas and think outside of the box, but seems to be less strongly related to the other factors (p. 2510).

Although we regard the FS as a valuable tool to explore rigidity more systematically, it is unclear whether the different factors identified by Strang and colleagues track a unitary phenomenon, or rather shed light on different facets of rigidity that may ultimately cluster together and influence one another in different ways. This ambiguity becomes clearer when we look at the correlation among the four main factors (Routines/rituals, Transitions/change, Special Interests, and Social Flexibility). While some of them are only weakly correlated with one another (Special Interests and Social Flexibility), others exhibit a strong correlation but also a good deal of conceptual overlap. For instance, Routines/rituals and Transitions/change correlate strongly but also exhibit significant overlap in terms of the items they include. The Routines/rituals factor includes several references to *resistance to change* (e.g., “requires specific routes to familiar destinations”), while the Transitions/change factor lists *insistence on sameness*, an item that is usually included in descriptions of routines and ritualized behavior. Similarly, the Social Flexibility factor includes clear references to *executive functioning* and thus to the Transitions/change domain—e.g., “difficulty taking turns”. In sum, the four factors are not conceptually independent from one another, and this might partially explain the observed correlations. Despite the guiding assumption that the different dimensions of rigidity tested are all expressions of cognitive inflexibility, the factors developed by Strang and colleagues might thus not track a unitary phenomenon. To be clear, though, the FS is the most valuable tool we currently possess to explore the different rigidity facets we distinguish above. As such, it constitutes a helpful starting point to collect data about how different facets of rigidity may cluster together—or come apart—in different autistic individuals or subgroups along the autism spectrum.

In a more recent paper, Ozsivadjian et al. (2) explore CF in connection with externalizing (aggressive or outburst behaviors, and irritability) and internalizing (anxiety and low mood) behavior in autistic individuals. Although the paper focuses on CF as a predominantly cognitive mechanism, Ozsivadjian and colleagues report that their clinical experience has prompted them to adopt a broader outlook on this notion, one including behavioral and social aspects such as inflexible rule-following, reduced tolerance of uncertainty, and less flexible problem-solving. In this study, they see CF as a factor that importantly predicts the insurgence of externalizing symptoms: they suggest, for instance, that difficulties in problem-solving and generating alternative strategies, i.e., executive functioning issues, would be responsible for negative reactions to adverse events and thus for externalizing behavior. They also suggest a more indirect path from lack of CF to internalizing symptoms *via* intolerance of uncertainty. As they put it, cognitive inflexibility may exacerbate uncertainty in the social domain, given that inflexible people would have trouble predicting how others will behave, thereby enhancing anxiety. Despite the broader notion of CF adopted, these researchers thus still endorse a CF-first approach, as they propose a direction of explanation that goes from cognitive functioning (e.g., problem-solving) to behavior (i.e., internalizing and externalizing symptoms).

Yet, assuming that all the different rigidity facets relate to cognitive inflexibility, however broadly the notion is understood, is unmotivated. Research does not support the claim that cognitive inflexibility has a cascade effect that could explain the appearance of other rigidity facets [see for instance (32)]. Actually, as we show in the next section, cognitive inflexibility can be conceptually dissociated from other rigidity facets. On the one hand, cognitive inflexibility can be present while most of the other facets are absent. On the other hand, while cognitive inflexibility might potentially explain other rigidity facets, equally plausible alternative hypotheses are available as to why such aspects may cluster together in autism. More research is needed in this area before any of these hypotheses can be legitimately assumed. In what follows, we set out to show that this is the case and we propose some ways in which research and interventions on rigidity could move forward.

## Dissociations among facets of rigidity and the challenge to CF-first approaches

To better understand and ultimately explain rigidity it would be important to find out whether some facets of this construct may be pried apart from one another. If this is so, as we set out to show, assuming a unifying picture of rigidity through the CF-first approach is problematic. The dissociations among facets of rigidity have relevant consequences for clinical practice, given that we may investigate rigidity as a transdiagnostic trait whose subcomponents cluster together differently in different conditions [see Servaas et al. (33)], and test different etiological explanations about the co-occurrence of different facets.

In this section we first show that studies such as Strang et al. (5) point to there being dissociations among different facets of rigidity. Then we show that these facets are also *conceptually* dissociable, and in particular that they are dissociable from CF functioning. Finally, we challenge the CF-first approach by offering some alternative explanations about why different facets of rigidity may cluster together in the autism spectrum.

Going back to the work conducted by Strang and colleagues, we may find some evidence for dissociations between different facets of rigidity. Setting aside Generativity (weakly correlated with the other factors), one example of dissociation concerns Special Interests (what we call “Fixed/restricted interests” above). Indeed, their findings suggest that Special Interests would be less pronounced in the female population, which implies that they would be dissociated from the remaining three factors. Similarly, Strang and colleagues discuss the possibility that Special Interests may be more acute in older autistic people (12), who, on the other hand, experience fewer inflexibility issues overall. Moreover, as we mention above, some of the FS scale factors conceptually overlap with items that refer to other constructs (especially to CF constructs). Given that correlations between the factors are already of moderate size [(5), p. 2510], it is predictable that they would be even weaker if overlapping items were to be factored out. That is, depending on how the factors are construed, even more dissociations are likely to emerge. Further evidence concerning dissociations between rigidity facets may be found in Kelly and Reed (32) recent study, where they suggest that weak central coherence and cognitive flexibility are dissociated from one another in the autistic population (though they found some correlation between weak central coherence and stereotypical behavior).

We now propose to discuss, at a more general level, whether and how the facets of rigidity discussed above may be *conceptually* dissociated (i.e., defined in such a way that conceptual connections between them are minimized), while offering some evidence in support of actual dissociations. We take this to be a preliminary step toward a more systematic research on rigidity facets.

(1) ***Fixed/restricted/special interests***: Having fixed interests is a facet of rigidity that may be closely related to insistence on sameness. Actually, within the items codified by ADOS-2, repetitive or circumscribed interests appear jointly in the same score. Also, in a review of restricted interests, Kimhi (34) talks about the construct as follows: “Restrictive interests may be repetitious (i.e., spinning a wheel) and/or limited in scope or range (i.e., a narrow or limited range of items that hold the individual’s interest)”. However, the fact that restricted interests may result in repetitive behavior (because the person is interested in a certain activity) does not imply that such repetitive behavior exhibits IOS, depending on how this latter notion is construed. In principle, it is possible to distinguish between repetitive behaviors prompted by fixed interests from repetitive behaviors, such as routines, that do not stem from any particular interest in seeing how the routine unfolds. A child who wants to be driven to school in a certain way, for instance, may be interested in seeing the same streets, corners, lights, etc., or may just find comfort in following the routine regardless of what the routine itself implies. This means that, in principle, the same pattern of behavior could be either a fixed interest pattern or an IOS pattern. So, depending on how we characterize IOS, fixed interests and IOS can be pried apart. This dissociation can be made more apparent by comparing autism with obsessive compulsive disorder (OCD), where insistence on sameness and ritualized patterns of behavior also feature prominently. However, these features in OCD appear to be strongly connected with anxiety and unrelated to fixed or restricted interests. In fact, compulsions in OCD are usually found to be ego-dystonic, that is at odds with one’s consciously endorsed beliefs or preferences (35).
(2) ***Insistence on sameness:*** This facet can be thought of as referring to scheduling activities in routines and rituals, or ordering objects in particular ways, irrespective of the reaction one might have following the failure to comply with such routines and rituals. In fact, such a reaction arguably depends on other factors, such as whether the relevant change is experienced as an improvement or a deterioration of one’s current situation. This means that there is room for an IOS notion that neither implies adverse reactions to changes nor repetitive behaviors prompted by restricted interests, but rather signals a preference toward a stable and structured environment (36). While IOS is not typically used this way, we think it is advisable to have a more regimented notion of IOS so that it does not overlap with other constructs, such as fixed interests or cognitive flexibility.
(3) ***Intolerance of uncertainty:*** This facet seems to refer to a cognitive trait that has behavioral implications—a person exhibiting intolerance of uncertainty may be uncomfortable with situations that can be thought of in different ways or from different perspectives, or with situations that are not well-defined. Such situations undoubtedly generate anxiety in many autistic people. As characterized, intolerance of uncertainty seems to relate to black-and-white mentality, maybe as two sides of the same coin; however, in principle, black-and-white mentality can appear without intolerance of uncertainty. This construct, also known as splitting or dichotomous thinking (37), is related to several mental conditions, such as depression, borderline personality disorder, and eating disorders (38) where the profiles tend to be different from those on the autistic spectrum.
(4) ***Black-and-white mentality:*** This facet is also conceptually close to strict adherence to rules: even though black-and-white mentality is a broad cognitive feature, it suggests intolerance to exceptions. However, strict adherence to rules can also appear without black-and-white mentality, as the former may be a response to social situations that the person is not able to navigate by herself. At a funeral, for instance, people may follow rules strictly because they simply do not know how to behave otherwise; no black-and-white mentality seems to be involved. Similarly, in camouflaging experiences (39), people also seem to follow rules to rigidly interact with others without thereby exhibiting black-and-white mentality.
(5) ***Literalism:*** Inflexibility toward the uses of language that go beyond literal meaning has been linked to cognitive flexibility (27) and could be also related to intolerance of uncertainty, weak global coherence (40), or strict adherence to rules (22). However, all these are etiological accounts of literalism. As such, literalism is conceptually independent from other facets as it refers to how language is processed. Moreover, some degree of literalism (or at least issues with figurative language) also seems to appear in developmental language disorders (41), plausibly linked to lack of complete development of structural aspects of language. Similarly, literalism appears in neurotypical development at around 5 years of age, where a U-shaped development from flexibility to rigidity (at 3 years of age) and then back to flexibility (at 6 years of age) is observed (42).
(6) ***Weak Central Coherence:*** Focusing on details without capturing the global gist of a situation or task is another cognitive characteristic that, on the face of it, appears unrelated to the other rigidity facets, although it may have a causal impact on some of them. Take IOS: if subjects have problems with generalizations and with finding similarities between different situations, they may have a tendency toward doing things always the same way. Problems with weak central coherence may be also related to scarce abilities in switching the attentional focus from one thing to another. In any case, weak central coherence, although probably characteristic of autism, is a cognitive trait that appears to be unrelated to the other rigidity facets even in its behavioral expression—e.g., difficulties in narrative production or comprehension.
(7) ***Task-switching:*** The difficulty in switching from one task to another is different from the difficulty in abandoning the task that one has begun. Many autistic people tend to get absorbed by the task they are engaged in and keep going until it is completed. This pattern of behavior may be an expression of intolerance of uncertainty, rather than a task-switching issue *per se*. In fact, other conditions such as schizophrenia or attention deficit hyperactivity disorder (ADHD) exhibit a marked impairment in terms of task-switching as measured through the Wisconsin Card Sorting Task (43). Yet, in both these cases cognitive flexibility functioning—and executive functioning difficulties more generally—fail to result in a rigid behavioral profile similar to the one found in autism.

Despite these conceptual dissociations, many of these rigidity facets may appear correlated in empirical studies on autism and thus be interconnected as a matter of fact. Actually, as we discuss above, the CF-first approach assumes that they are all strongly influenced by executive functioning. It is surely tempting to link CF problems (or executive functions more generally) to most—if not all—rigidity facets by thinking about them as cascade effects of executive dysfunctions, even when different rigidity facets have been characterized in ways that do not include any reference to cognitive flexibility. Generally, people are said to be flexible when they can adapt their behavior to the changing demands of the environment. To be able to do that, one may say, they have to be able to consider the same thing or event in alternative ways, to follow different rules on different occasions, to consider more nuanced possibilities as opposed to extreme ones, etc. In sum, these different forms of flexibility seem to require a functioning executive system. Then, lacking the possibility to do such things, people may prefer to adopt more rigid behaviors.

However, the CF-first approach is only *one plausible explanation* of why some facets of rigidity come together, and it is still unclear how the approach would explain why they at times dissociate. We contend that other explanatory hypotheses may be at least equally plausible: in what follows we consider one of them, which we dub the “Social-first approach”. In difficult social situations, most people experience social awkwardness and at the same time exhibit at least some forms of rigidity: strict adherence to rules, intolerance of uncertainty, routines and rituals, and possibly also difficulties in switching from one task to another. People tend to become rigid (even physically) when they experience overwhelming social situations. So, it is plausible to think that problems about navigating the social world should have a cascade of effects on various dimensions of flexibility. If some individuals have problems understanding the social world, they will likely follow rules (not being able to predict when such rules admit exceptions) and they will also stick to conventions when trying to understand what others say. Problems with social interactions also generate anxiety, eventually leading to social anxiety disorders (44, 45),^8^ which may make people look for comfort in routines and rituals and render them particularly anxious about uncertainty.^9^ This may in turn have an impact on developing black-and-white mentality.^10^ Even task-switching may be impaired under social stress (46). In other words, a Social-first approach appears *prima facie* as reasonable as a CF-first approach to explain why several different facets of rigidity cluster together in autism.

Moreover, social motivation theories (47), according to which autism is characterized by a lack of social motivation, are also able to predict rigidity on various fronts. As described by the authors, social motivation is characterized as a set of psychological dispositions and biological mechanisms that orient individuals toward the social world, toward seeking and finding pleasure in social interactions and toward maintaining social bonds. If some people are not motivated—or simply unable—to socially interact, but they are forced to do it, they will probably also develop social anxiety, and along with it, intolerance of uncertainty, black-and-white mentality, and so on.

Now, although cognitive explanations of rigidity are still mainstream in several subfields within autism research, other alternatives have been proposed. Among them, it is worth mentioning predictive processing accounts of autistic traits (48, 49). According to these accounts, rigid thinking and behavior in autism would be the consequence of a specific way of processing information, where incoming input—sensory or otherwise—is assigned more weight than prior predictions. For instance, Pellicano and Burr (50), probably the first attempt at accounting for autistic symptoms from a predictive processing perspective, explain hypersensitivity as caused by “hypo-priors”—i.e., a diminished confidence in the brain’s own predictions as compared to the neurotypical brain. Some researchers connect this processing style with experiencing the world as being filled with error and uncertainty. As a consequence, they cash out some rigidity facets—such as insistence on sameness and repetitive behavior—as “attempts to provide a reassuring sense of predictive success” [(48), p. 649]. More generally, rigid thinking and behavior, including the reliance on routines and the strict adherence to rules, would emerge as a response that provides some reassurance in the face of a world filled with error and uncertainty (51). In sum, the preference toward structure and stability that many autistic people experience can be related to a diminished confidence in priors. The world gets chaotic easily, but it gets less overwhelming if it is made simple through increased structure, be it because the environment is arranged so that one’s predictions are successful (e.g., you rely on routines or schedules to know more confidently what is coming next), or because an external agent boosts confidence in one’s predictions, thereby lowering uncertainty as a result (e.g., another person tells you in detail what is going to happen next).

It is interesting to note that, from the point of view of predictive processing frameworks, Intolerance of Uncertainty is explained in terms of higher *uncertainty* instead of higher *intolerance of uncertainty* with respect to neurotypicals (19). We may distinguish between the two as follows. Intolerance of uncertainty would imply that two individuals, A and B, hold the same probability assignments, but display different cognitive, emotional, and behavioral reactions. In other words, A tolerates the same degree of uncertainty less easily than B. For example, neither A nor B know whether they will be able to catch the next flight, but they both know that it is highly likely. Yet, A gets more anxious than B. Higher uncertainty, by contrast, would imply different probability assignments that generate different cognitive, emotional, and behavioral reactions. In this case, A and B’s degrees of uncertainty are different to begin with: for instance, A might assign lower probability to (or be less confident about) each of the relevant predictions that compose the belief: “We are going to catch the next flight”. If confronted with each of these relevant predictions piece by piece, A would thus be more likely to experience higher uncertainty along the way—e.g., to answer “I don’t know” more often than B when confronted with questions about the upcoming flight.

Taking stock: if we aim at explaining why, in a number of cases still to be analyzed more thoroughly, different facets of rigidity come together, alternative accounts such as social accounts or predictive processing accounts, seem as valuable, illuminating, and capable of providing explanations or explanatory schemas as CF-first explanations. That is, such accounts constitute viable alternative etiological approaches to rigidity *vis a vis* the dominant CF-first approach. Is there a reason to prefer one over the other(s)? At the present stage of research, we believe there is not. We take it that all these approaches—as well as others not discussed in detail here—are only *prima facie* plausible explanations of a complex phenomenon. Before we try to adjudicate among these and other explanations, we need to be able to characterize each rigidity facet more precisely, and to know whether it is a valid independent construct that may be operationalized successfully.

## Final remarks and future directions

Clinical diagnoses of autism sometimes make unspecific reference to “rigid thinking” or “rigid behavior.” It is commonplace for professionals working on autism or with autistic people to highlight rigidity as a characteristic trait, and the notion is also widely discussed in first-person accounts. In order to build a more precise approach to rigidity and its causes, we propose to proceed through the following steps. First, we need to identify more precise and operationalized notions of the different facets discussed, while being cautious not to mix them conceptually. We attempted to make some initial progress in this direction through our discussion of the different facets of rigidity: ultimately, the goal would be to achieve construct *validity* for each of these notions. Second, further research is needed on the distribution and clustering of the different rigidity facets in the autistic population [see (32) for a recent attempt]. At the moment, we do not know exactly how often facets (or sub-clusters of them) come together, nor how often autistic people exhibit just a few of these traits in isolation. Third, we need to propose and assess etiological hypotheses for the co-occurrence of different facets of rigidity, while testing them against cases where they do not come together, seeing how the various competing hypotheses account for such dissociations. In this spirit, we introduce the predictive processing and the social approaches to rigidity as plausible and testable alternative hypotheses. We believe the steps described above are necessary to reach a comprehensive approach to rigidity in autism.^11^

A systematic and comprehensive study of the rigidity construct is therefore crucial to acquire better knowledge and provide better treatment, as well as more individualized support to autistic individuals. This is bound to be relevant in intervention, where identifying different facets of rigidity, observing how (and how often) they cluster together, and measuring them effectively may have a significant impact. For instance, if we were to find that several rigidity aspects boil down to cognitive issues related to task-switching, then interventions on transitions and scheduling would be particularly important (52). Alternatively, the idea that treating circumscribed interests has a direct impact on externalizing behavior (2) could be reinforced, or that intervention on cognitive flexibility and planning can contribute to improving social skills (53). On the other hand, if various rigidity facets are responses to social difficulties, intervention on social settings would be the relevant target. Similarly, if rigidity facets in autism relate to low confidence in predictive priors, or to some other difficulty in adjusting predictions to volatile environments, intervention should be focused on dealing with uncertainty, as well as on reducing environmental volatility. It is also important to bear in mind that interventions have to occur simultaneously in different contexts, since one of the peculiarities of rigidity is that learning is often context-bound—i.e., abilities acquired in a context are not easily transferred to other contexts.

Methodologically, our reflection on rigidity may be taken as the starting point to develop new assessment tools, besides the Flexibility Scale, such as questionnaires or semi-structured interviews for families, teachers, caregivers and students. Increased attention to multiple facets of rigidity and how they cluster together would allow for more individualized plans, as well as for more specific interventions targeted toward the aspects of rigidity experienced as more challenging. Such a finer-grained assessment of rigidity would ideally take into account both quantitative measures (e.g., frequency and duration of inflexible behavior) and the impact on the social and educational context experienced by each individual.

After having properly characterized and operationalized the various rigidity facets, and having garnered more knowledge on how they cluster together and dissociate from one another, it will also be easier to pin down some rigidity facets as possible sources of *strength* in specific domains and contexts (54). In professional settings, for instance, some aspects of rigidity—such as attention to details, tolerance for repetitive tasks, and special interests—are bound to be advantageous (55). Similarly, there is evidence that a more structured learning environment and the incorporation of special interests into classroom practices positively impacts learning (56). As several researchers have recently suggested (4, 36), intervention should not necessarily be targeted at diminishing rigidity *per se*, but rather at facilitating adaptation while devising strategies to navigate challenging situations (e.g., shifts, stress-inducing tasks, etc.). In this paper we mostly focused on the *challenges* raised by rigidity in autism, but it is also important to devise interventions that go in the direction of looking for co-adaptations between individuals and their environment that acknowledge and possibly take advantage of some rigidity facets.

## Data availability statement

The original contributions presented in this study are included in the article/supplementary material, further inquiries can be directed to the corresponding author.

## Author contributions

All authors listed have made a substantial, direct, and intellectual contribution to the work, and approved it for publication.

## Footnotes

1 In line with the autism rights movement and neurodiversity proponents, in this paper we use the label ‘autism’ or ‘autism spectrum’ interchangeably, instead of ‘autistic spectrum *disorder’* (ASD) or ‘autistic spectrum *conditions*’ (ASC). However, we acknowledge that ASD is still widely used as a diagnostic label (1), and that most of the studies discussed here employ this terminology. Moreover, following the results reported in recent qualitative analyses of linguistic policies surrounding autism (57, 58), we use identity-first language in our discussion (i.e., “autistic person/individual” instead of “person with autism”).

2 IOS is also discussed by autistic people in first-person accounts: some describe their carefully crafted routines as a combination of journals, diaries, and to-do lists; others talk about the need of having each day structured in exactly the same way; and still others describe a range of repetitive behaviors such as stimming, hand-flapping, arranging objects or clothes in a particular way, etc. [See (59, 60)].

3 First-person accounts describe coping mechanisms and strategies to minimize the amount of uncertainty in everyday life: this at times translates into experiencing some everyday tasks or actions—e.g., house chores—as challenging because of their paradigmatically “unfinished” character (61, 62).

4 Autistic writer and activist Jorik Mol brings up this aspect when exploring his relationship with work and productivity: “I only want to produce at the highest level and want progress to be at a clean, 90 degree angle […] That’s how my autistic black-white thinking combines with my lack of intuition about how much energy I’ve got on a day. I don’t yet have the skill to be ok with having a bad day. I see it as a personal failure” (59).

5 To express this notion some autistic people compare themselves to a car driving on cruise control on the highway at exactly the speed limit. Although technically that is what everyone is supposed to do, there are a million scenarios—especially in social settings—where people are required to flexibly respond to unpredictable changes, and thus metaphorically “slow down” or “speed up” when needed (63).

6 First-person accounts underscore such a difficulty with generalization: “Because I have trouble generalizing from one situation to another, I won’t foresee the new mishap based on a similar one from the past” (60).

7 As the autistic writer and cartoonist Erin Human vividly puts it in a comic strip: “When I’m focused on something, my mind sends out a million tendrils of thought. Expands into all of the thoughts and feelings. When I need to switch tasks, I must retract all of the tendrils of my mind. This takes some time. Eventually I can shift to the new task. But when I am interrupted or must shift abruptly, it feels like all of the tendrils are being ripped out. That’s why I don’t react well” (64).

8 Social anxiety does not imply social skill deficits, as it can be triggered even in cases of socially ambiguous or threatening situations. However, poor social skills are related to experiencing social anxiety [see (44)].

9 See again Morrison and Heimberg (44) on safety behaviors. Note, however, that intolerance of uncertainty may also have a causal role in generating social anxiety [see (65)].

10 It is not clear whether black-and-white mentality may be an effect of anxiety and uncertainty (45), or rather a causal factor that increases both anxiety and intolerance of uncertainty (66). It is likely that both variables enter into feedback loops.

11 By adopting a transdiagnostic approach to rigidity, it might also be easier to associate different clusters of rigidity features with different conditions or disorders and see how they interact. One interesting case would be to compare rigidity profiles between autism and OCD, as these two conditions share a number of features (e.g., routines, ritualized patterns of behavior) as well as significant differences—e.g., the emotional valence associated with the performance of rituals (35).
